# Proteome profiling of plant Cajal bodies uncovers kingdom-specific components

**DOI:** 10.1093/nar/gkag867

**Published:** 2026-09-18

**Authors:** Yu Zhou, Huang Tan, Axel Giudicatti, Angela Vicente-Luque, Marvin Weis, Lena Wehinger, Marina Schreidl, Irina Droste-Borel, Boris Macek, Kenneth Wayne Berendzen, Rosa Lozano-Durán

**Affiliations:** Plant Biochemistry Department, Center for Plant Molecular Biology (ZMBP), Eberhard Karls University Tübingen, Tübingen,D-72076, Germany; Guangdong Provincial Key Laboratory for Plant Epigenetics, College of Life Sciences and Oceanography, Shenzhen University, 518060, Shenzhen, China; Plant Biochemistry Department, Center for Plant Molecular Biology (ZMBP), Eberhard Karls University Tübingen, Tübingen,D-72076, Germany; Plant Biochemistry Department, Center for Plant Molecular Biology (ZMBP), Eberhard Karls University Tübingen, Tübingen,D-72076, Germany; Plant Biochemistry Department, Center for Plant Molecular Biology (ZMBP), Eberhard Karls University Tübingen, Tübingen,D-72076, Germany; Plant Biochemistry Department, Center for Plant Molecular Biology (ZMBP), Eberhard Karls University Tübingen, Tübingen,D-72076, Germany; Plant Biochemistry Department, Center for Plant Molecular Biology (ZMBP), Eberhard Karls University Tübingen, Tübingen,D-72076, Germany; Plant Biochemistry Department, Center for Plant Molecular Biology (ZMBP), Eberhard Karls University Tübingen, Tübingen,D-72076, Germany; Proteome Center Tübingen, Eberhard Karls University Tübingen, Tübingen, D-72076, Germany; Proteome Center Tübingen, Eberhard Karls University Tübingen, Tübingen, D-72076, Germany; Cluster of Excellence GreenRobust, Eberhard Karls University Tübingen, Tübingen, D-72076, Germany; Plant Biochemistry Department, Center for Plant Molecular Biology (ZMBP), Eberhard Karls University Tübingen, Tübingen,D-72076, Germany; Cluster of Excellence GreenRobust, Eberhard Karls University Tübingen, Tübingen, D-72076, Germany; Plant Biochemistry Department, Center for Plant Molecular Biology (ZMBP), Eberhard Karls University Tübingen, Tübingen,D-72076, Germany; Cluster of Excellence GreenRobust, Eberhard Karls University Tübingen, Tübingen, D-72076, Germany; Department of Plant Virology, Max Planck Institute for Plant Breeding Research, Cologne, D-50829,Germany

## Abstract

Membraneless organelles (MLOs) in eukaryotic cells, such as the nuclear Cajal body (CB), have been notoriously difficult to biochemically isolate due to their dynamic and delicate nature. CBs, formed via liquid–liquid phase separation, mediate RNA processing and ribonucleoprotein assembly, essential for nuclear functions. In plants, this challenge has prevented any direct molecular characterization to date. Here, we report a successful isolation of plant CBs, achieved through a combination of biochemical purification and fluorescence-activated nuclei and particle sorting, enabling a previously unattainable biochemical access to this organelle. Mass spectrometry analysis defined a high-confidence set of plant CB-associated proteins, identifying 15 and 79 proteins uniquely localized and enriched in CBs, respectively, of which 18 are also labelled by CB markers in proximity labelling assays. While a partial overlap with known animal CB proteins exists, our data suggests a partially divergent composition of CB-associated proteins across kingdoms. This work breaks a major technical challenge, enabling detailed characterization of plant CBs and establishing a versatile platform adaptable for the isolation and molecular dissection of other MLOs.

## Graphical Abstract

**Graphical Abstract:**
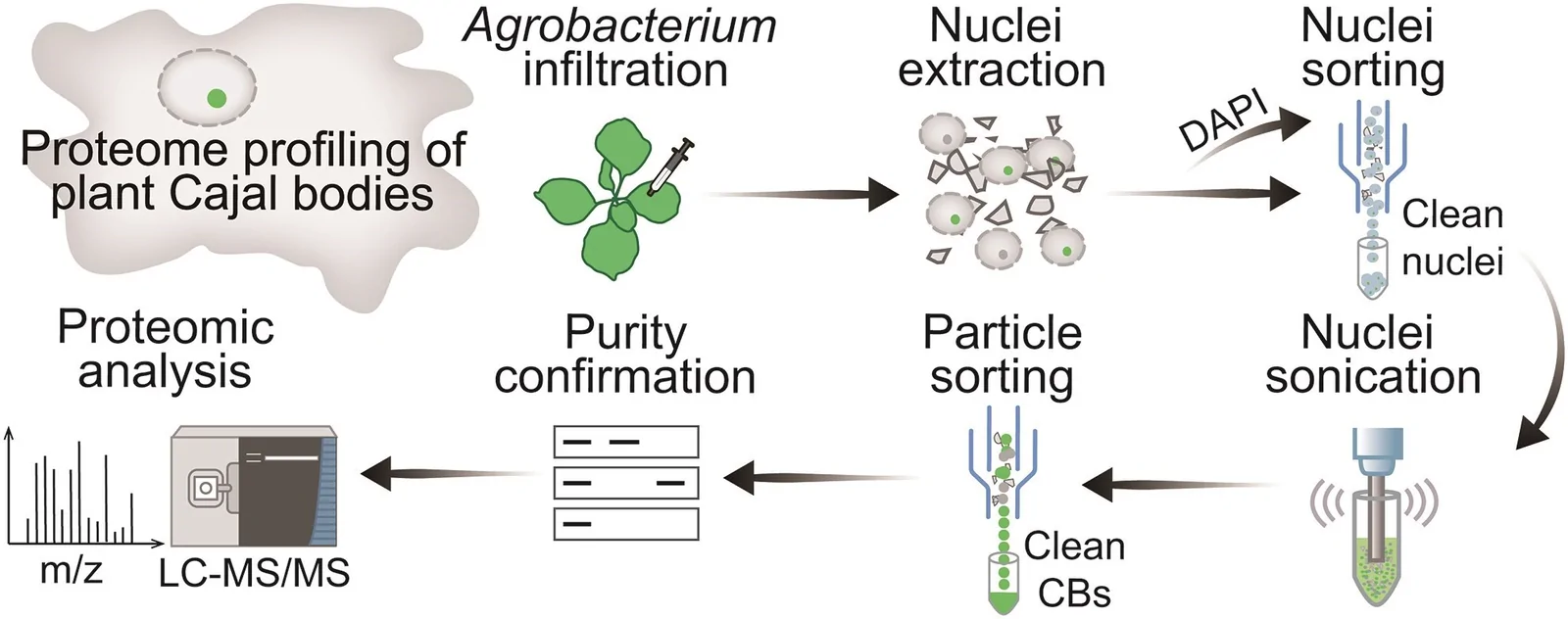

## Introduction

Heterogeneity and compartmentalization are fundamental features of living cells, with cellular activity spatially distributed across different microenvironments. Beyond the hallmark eukaryotic membrane-bound organelles, membraneless organelles (MLOs) and biomolecular condensates have emerged in recent years as widespread cellular structures with essential biological roles. Nevertheless, the full functionality of such molecular assemblies remains in most cases to be determined. A first necessary step towards a complete understanding of these structures is the definition of their molecular components; this is, however, challenged by their dynamic nature, their potential compositional complexity, and, importantly, the difficulties in their isolation.

One such MLO is the Cajal body (CB). The CB is a nuclear condensate with a diameter of 0.15–2 µm, initially described in vertebrate neurons by Santiago Ramón y Cajal in 1903, found in animals and plants and believed to form via liquid–liquid phase separation (LLPS). These bodies are present in varying numbers per nucleus, form at the sites of transcription and processing of small nuclear/nucleolar RNAs (SnRNA/SnoRNA), and play a role in the biogenesis and assembly of ribonuclear protein (RNP) particles [1]. The defining component of the CB is coilin, a conserved scaffolding protein required for formation of this MLO [2]. Coilin undergoes LLPS *in vivo* through its intrinsically disordered region domain [3]. Beyond coilin, however, our knowledge on CB composition is limited. Recent studies have characterized the proximal proteome of coilin by proximity labelling (PL) in animal cell cultures [4–6]. Nevertheless, the resulting datasets are likely to capture a partial CB proteome, restricted by the bait proteins in a certain spatial domain. The characterization of the CB proteome would require purification of this organelle, a non-trivial task considering its membraneless nature.

Strikingly, the compositional information on the plant CB is even more scarce. Filling this knowledge gap is particularly pressing given the relevance of CBs for plant biology [7] and the hints at potential fundamental differences between animal and plant CBs. For example, plant CBs harbor components of the plant-specific RNA-directed DNA methylation (RdDM) pathway [8] and have emerged as convergent targets of viruses [7, 9]. In this work, we provide the first proteomic characterization of plant CBs using the newly developed tandem fluorescence-activated nuclei and particle sorting (FANS-FAPS) approach, identifying previously unknown components. In addition, some of the coilin proximal proteins identified in human cell cultures lack obvious orthologs according to our ortholog analysis [4, 5], suggesting that some molecular components associated with CBs may have diverged between kingdoms.

## Materials and methods

### Plant material and growth conditions

*Nicotiana benthamiana* plants were grown in a controlled growth room under long-day conditions (16 h light and 8 h dark) at 25°C.

### Plasmids construction

All primers used in this study are summarized in Supplementary Table S7.

For construction of the CB marker used for FAPS, the *pRPS5A* promoter was adapted from the GreenGate system [10], with modifications to the overhang sequences. The *pRPS5A* and *pNbCoilinA* promoter and *NbcoilinA* (Niben101Scf04802g01014) were assembled into Level II vector from the GoldenGate-based toolkit [36] via *Bsa*I cut-ligation, along with the following modules: DE-GFP/DE-mcherry, EF-nosT, and FG-dummy. The *Arabidopsis thaliana* gene *AtPhyB* (AT2G18790) and *N. benthamiana* genes *NbU2A* (Niben101Scf02581g05011), *NbSmB* (Niben101Scf01968), *NbNop10* (Niben101Scf02243g01029), *NbL7A*e (Niben101Scf03147g07006), *NbSDN3* (Niben101Scf12361g03004), *NbSART3* (Niben101Scf00705g05008), *NbNTP4* (Niben101Scf03208g00007), *NbWRAP53* (Niben101Scf07372g00012), *NbTAF15* (Niben101Scf04103g10003), *NbSKP1* (Niben101Scf02378g02004), and *NbRBM1* (Niben101Scf02797g00005) were cloned into Level II vector at the CD position using *Bsa*I/*Esp3I* cut-ligation, together with modules AC-p35S, DE-GFP, EF-nosT, and FG-dummy. Gateway-compatible constructs for *NbSPL1* (NbS00001319g0219), *NbSLU7* (Niben101Scf04193g06007), *NbSASF4* (Niben101Scf04925g00004), *NbCWF19* (Niben101Scf12847g00018), *NbRP* (Niben101Scf02001g01004)*, AtU2B*’’ (At2g30260), *AtSmD3* (At1g76300), *NbcoilinA*, and *NbcoilinB* (Niben101Scf04939g02015) were generated by cloning into the destination vectors pGWB505, pGWB506, pGW-GOI-TurboID, and pGW-GOI-TurboID-GFP [37].

### Agrobacterium-mediated transient expression

*Agrobacterium tumefaciens* strain GV3101(pMP90) was used for delivery of plasmid constructs into *N. benthamiana* leaves as previously described [37].

### Cajal body isolation and fluorescence-activated particle sorting

Leaves from 4-week-old *N. benthamiana* plants transiently transformed with pRBS5a*:NbcoilinA-GFP* (NbcoilinA-GFP) or an empty vector (NC) were crosslinked in a fixation buffer containing 10 mM Tris–HCl, 10 mM ethylenediaminetetraacetic acid (EDTA), 100 mM NaCl, and 0.1% Triton X-100, supplemented with 1% formaldehyde. Vacuum infiltration (50 psi) was applied for 10 min twice. Crosslinking was quenched by vacuum infiltration with 125 mM glycine for 10 min. Tissue was then homogenized using a blender (SEVERIN, UZ3861) in lysis buffer (15 mM Tris–HCl, 2 mM EDTA, 80 mM KCl, 20 mM NaCl, 0.5 mM spermine, 15 mM dithiothreitol, 10 mM protease inhibitor, and 0.1% Triton X-100) at 260 W in 30-s on/off mode for a total of five cycles. The homogenate was centrifuged at 800 × *g* for 10 min at 4°C. The pellet was then resuspended in suspension buffer (20 mM Tris–HCl, 1.5 mM MgCl_2_, 10 mM KCl, 15 mM dithiothreitol, and 10 mM protease inhibitor for nuclei sorting (see below). Sorted nuclei were sonicated using a SONOPULS Ultrasonic Homogenizer (BANDELIN) at 40% power in 15-s on/off cycles six times. The lysate was centrifuged at 800 × *g* for 10 min at 4°C, and the resulting supernatant containing released CBs was subjected to CB sorting (see below). Nuclear fractions were collected at this step from both NbcoilinA-GFP-expressing samples (Input) and EV-expressing samples (NC), then freeze-dried using an Alpha 1–4 LSC vacuum dryer (CHRIST) at –60°C for 48 h. The resulting powder was stored at room temperature until further use. Sorted CBs were pelleted by ultra-centrifugation at 42 000 × *g* for 45 min. The pellet was stored at –80°C until further use. Based on empirical recovery estimates, ~5 g of Agrobacterium-infiltrated *N. benthamiana* leaf tissue yielded 10 million nuclei, from which 2–3 million CBs could be isolated.

Nuclei and CB sorting were performed using a MoFlo XDP cell sorter (Beckman Coulter) equipped with a 70 µm nozzle, operating at a sample/sheath pressure differential of 61/60 psi. Phosphate-buffered saline (pH 7.0) was used as the sheath fluid, and sorting was conducted in Purify 1 Drop mode to ensure high purity. For nuclei sorting, 0.1 µg/ml DAPI (4′,6-diamidino-2-phenylindole; Invitrogen) was used to stain nuclei and excited with a 15 mW, 375 nm laser in spherical focus mode. DAPI emission was collected using a 465/30 nm bandpass filter. GFP and side scatter were excited with a 75 mW, 488 nm laser using a cross-cylindrical focus. GFP emission was collected with a 534/30 nm bandpass filter, and side scatter with a 488/10 nm filter. Nuclei were identified as bright DAPI-positive populations compared to side scatter. Doublets and clumps were excluded by comparing DAPI peak height to area values. GFP-positive nuclei were confirmed by plotting DAPI versus GFP fluorescence and comparing to untransformed negative controls (NCs). CBs were identified as small, GFP-positive particles distinguishable from nuclei by their lower side scatter profile. Gating was optimized by comparing GFP versus side scatter plots between samples and NCs. To ensure precise gating during sorting, live acquisition was monitored by displaying the most recent 25 000 events.

### TurboID-based proximity labelling assays

Leaves from 4-week-old *N. benthamiana* plants transiently transformed with constructs to express TurboID-fused proteins or free TurboID were processed for PL assays as described [37] and subjected to liquid chromatography–tandem mass spectrometry (LC-MS/MS) analyses.

### Western blotting and RT-qPCR

**Figure 1.**
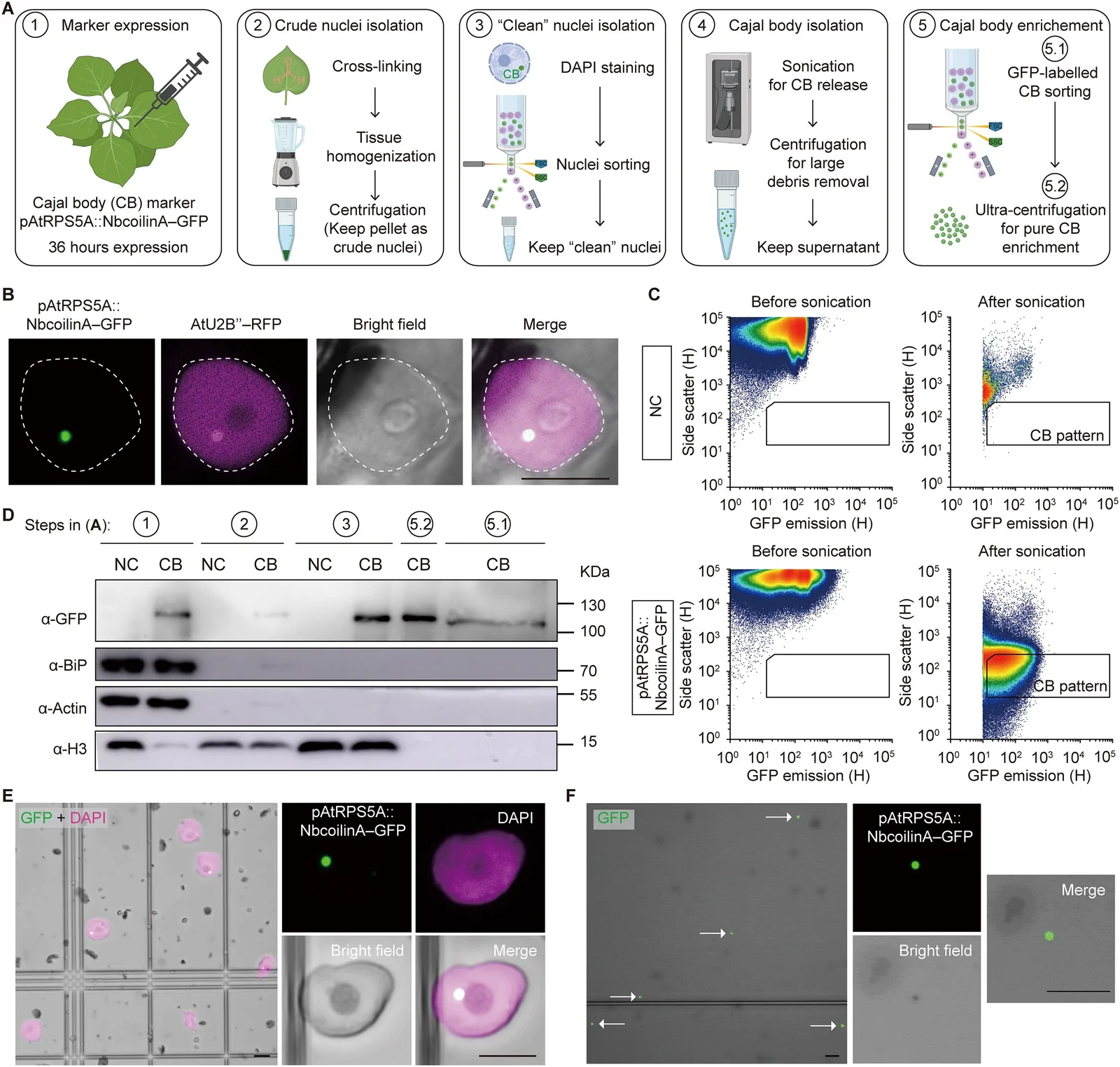
Isolation of CBs from *N. benthamiana* using fluorescence-activated particle sorting. (**A**) Schematic overview of the CB isolation workflow (created with BioRender.com). Leaves expressing a CB-marker (NbcoilinA-GFP) were crosslinked with formaldehyde. Crude nuclei were extracted and then sorted by their DAPI signal. Following sonication and centrifugation, the supernatant was used for sorting for CBs, and sorted CBs were collected by ultra-centrifugation. Leaves transformed with an empty vector were used as NC. (**B**) Confocal imaging showing the specific CB localization of NbcoilinA-GFP; AtU2B''-RFP is used as CB marker. Scale bar = 10 µm. (**C**) Representative flow cytometry plots of side scatter versus GFP emission used to define the CB gating strategy. GFP-positive particles present in the marker-expressing samples but absent in the NC were selected. H: height. (**D**) Immunoblot analysis of samples from each purification step from panel (A), showing enrichment of NbCoilinA-GFP (∼120 kDa) and absence of major organelle contaminants. Markers include BiP [endoplasmic reticulum (ER), 80 kDa], Actin (cytosol, 45 kDa), and Histone H3 (nucleus, 17 kDa). (**E**) Confocal images of sorted nuclei (‘clean nuclei’) stained with DAPI (magenta) and expressing NbCoilinA-GFP (green). Scale bars = 10 µm. (**F**) Confocal images of GFP-positive particles representing FAPS-isolated CBs. Arrowheads mark CB. Scale bars = 10 µm.

Western blot analysis was performed as previously described [37]. For the western blot in Fig. 1D, equal starting amounts of leaf material were used for CB and NC samples. Lane 1 contained total protein extracted from 0.5 g leaf tissue; lane 2 contained crude nuclei extracted from 0.5 g leaf tissue; lane 3 contained 0.5 million FANS-sorted nuclei; lane 5.1 contained 1 million sorted CB particles; and lane 5.2 contained 1 million sorted CB particles that were pelleted by ultra-centrifugation. The primary and secondary antibodies used are the following: goat anti-GFP (Sicgen, Catalog number AB0020-500, 1:15 000 dilution); mouse anti-HA (Sigma–Aldrich, H3663; 1:3000); mouse anti-Actin (Agrisera, AS21-461510, 1:2000); rabbit anti-BiP (Agrisera, AS09-481, 1:2000); rabbit anti-H3 (Agrisera, AS09-481, 1:5000); anti-mouse peroxidase (Sigma–Aldrich, A2554; 1:15 000 dilution); anti-goat peroxidase (Sigma–Aldrich, A8919, 1:20 000); and anti-rabbit peroxidase (Sigma–Aldrich, A0545, 1:15 000).

Nucleic acid isolation and reverse transcription quantitative polymerase chain reaction (RT-qPCR) were performed as previously described [38]. Primers used for RT-qPCR are listed in Supplementary Table S7.

### Liquid chromatography–tandem mass spectrometry analysis

Pellets collected after CB isolation were resuspended in 4× NuPAGE LDS sample buffer and heated at 95°C for 10 min to reverse formaldehyde crosslinking prior to sodium dodecyl sulphate–polyacrylamide gel electrophoresis (SDS–PAGE). In four independent biological replicates, 7.65 million, 8.9 million, 8.4 million, and 9.1 million particles were collected, respectively. Approximately 8 million isolated CB particles yielded 2.5 µg of total protein, estimated by Coomassie staining using HeLa protein standards. Proteins were separated by SDS–PAGE, followed by in-gel digestion [39]. Extracted peptides were desalted using C18 StageTips [40]. Equal peptide amounts were subjected to LC-MS analysis on a VanquishNeo nano-UHPLC coupled to Exploris 480 mass spectrometer via nanoelectrospray ion source (Thermo Fisher Scientific). Peptides were separated on a custom-packed nano-LC column (20 cm length, 75 µm inner diameter) containing 1.9 µm C18 ReproSil-Pur beads using a 49-min segmented gradient from 4% to 55% of solvent B (80% acetonitrile in 0.1% formic acid) at a flow rate of 300 nl/min. MS and MS/MS spectra were acquired in positive ion mode with a resolution of 60 000 for MS1 and 30 000 for MS2 scans. The AGC target was set to ‘standard’, and the maximum injection time was set to ‘auto’. Precursor ion selection was performed using a 1.3 Th isolation window, and the top 20 most intense precursors were selected for higher-energy collisional dissociation per scan cycle. Dynamic exclusion was set to 30 s. TurboID samples were analysed on an Easy-nanoLC coupled to a Q Exactive HF mass spectrometer (Thermo Fisher Scientific) using a 49-min gradient (10%–33%–50%–90% solvent B) at 200 nl/min. The top seven precursors were fragmented, with resolution set to 60 000 for both MS and MS/MS spectra and an AGC target of 3E6.

### MS data processing and downstream analysis

Raw MS data were analysed using MaxQuant software (version 2.2.0.0) [41], employing the integrated Andromeda search engine [42]. Spectra were searched against a *N. benthamiana* protein Niben101 database (Sol Genomics Network), supplemented with a database of 286 common laboratory contaminants. Sequences for NbcoilinA-GFP were added as custom entries to the search database. Default MaxQuant parameters were used, with intensity-based absolute quantification and label-free quantification (LFQ) enabled. The ‘match between runs’ feature was activated for samples within the same experimental group to improve peptide identification consistency.

Statistical comparisons were conducted in Perseus software (version 1.6.15.0) following log_10_ transformation of LFQ intensity values. Significance testing was performed using a two-sample *t*-test with permutation-based false discovery rate control set to 0.05 and *S*₀ = 0. Data were filtered to retain only proteins with at least two valid LFQ values in one of the comparison groups. The Niben101 ID was converted to TAIR10 ID at Niben2Arab. The TAIR10 IDs were used for Gene Ontology (GO) analysis, STRING analysis and ortholog analysis. For GO analysis, the reference protein set used for enrichment analysis consisted of all proteins detected by MS across all samples rather than the full *Arabidopsis* proteome, thereby accounting for MS detectability and sampling bias.

### Confocal laser scanning microscopy

Confocal imaging of the subcellular localization of GFP- or RFP-tagged proteins expressed in *N. benthamiana* epidermal cells was performed using a Zeiss LSM880 confocal laser scanning microscope. GFP was excited with a 488 nm laser and detected between 500 and 550 nm. RFP was excited with a 561 nm laser and detected between 570 and 630 nm. For nuclear staining, 0.1 µg/ml DAPI was applied and excited using a 405 nm laser, detected in the 420–470 nm range.

## Results

### A fluorescence-based method for isolation of CBs

To investigate the protein composition of plant CBs, we developed a method based on tandem FANS-FAPS to isolate this MLO from *N. benthamiana* leaves (Fig. 1A). To enable specific labelling of CBs, the CB scaffold protein NbCoilinA [10] was fused to GFP and expressed under either its native promoter, the ribosomal protein subunit 5a (pRPS5A) promoter [10], or the 35S promoter. NbCoilinA-GFP displayed a similar localization pattern when expressed under either its native promoter or the pRPS5A promoter, with the majority of nuclei containing a single CB (Fig. 1B and Supplementary Fig. S1A and B). Under these conditions, NbCoilinA-GFP specifically localized to CBs, as demonstrated by its co-localization with the established CB marker AtU2B″ [11]. In contrast, 35S-driven expression frequently resulted in multiple CBs per nucleus (Supplementary Fig. S1C). We therefore selected the pRPS5A promoter for subsequent experiments, as it provided improved fluorescence intensity for efficient FANS-FAPS sorting while largely preserving native CB morphology.

NbCoilinA-GFP was transiently expressed in fully expanded leaves of 4-week-old *N. benthamiana* plants. As a negative control for the entire isolation procedure, an empty vector lacking the GFP tag (NC) was used in parallel. Transient transformation with the empty vector resulted in no detectable GFP signal and was used to define the background for the isolation. Samples were harvested at 36 h post-infiltration and cross-linked with formaldehyde to preserve CB integrity during isolation. The tissue was further homogenized using a kitchen blender to release crude nuclei, which were then enriched by FANS following DAPI staining. FANS was initiated by triggering a fluorescence threshold for DAPI and plotting DAPI emission against the side scatter (SSC) (Supplementary Fig. S2A). Both nuclei from NbCoilinA-GFP and NC samples were identified as DAPI-positive populations with distinct scatter profiles. Singlet nuclei were sorted by plotting the DAPI emission height against its area, and both gates were used for sorting. To verify the presence of GFP-positive nuclei in the NbCoilinA-GFP sample, DAPI fluorescence was plotted against GFP fluorescence. While NC samples showed only background fluorescence, NbCoilinA-GFP samples contained a distinct GFP-positive nuclei population, confirming successful labelling of CB-containing nuclei.

The purified nuclei were disrupted by sonication to generate a nuclear extract, from which intact CBs were isolated using FAPS. GFP-positive particles were identified as small structures with lower SSC intensity than intact nuclei (Fig. 1C and Supplementary Fig. S3A–G), consistent with the expected size and complexity of CBs. Gating parameters were optimized by comparing GFP versus SSC plots across NbCoilinA-GFP and NC. This gating strategy enabled the reproducible isolation of a distinct and consistent population of GFP-positive particles in NbCoilinA-GFP expressing samples, which was absent in the NC. In contrast, several other nuclear proteins did not generate similarly distinct high-GFP particle populations within the same sorting window (Fig. S3C–G), suggesting that the observed FAPS pattern is characteristic of NbCoilinA-associated structures rather than a general feature of nuclei protein aggregates.

To further confirm that the FAPS-isolated particles were enriched in NbCoilinA-GFP, an immunoblot was performed using an anti-GFP antibody on equal amounts of protein from leaf tissue, crude nuclei, purified nuclei, and FAPS-isolated particles from samples expressing the NbCoilinA-GFP or transformed with the NC (Fig. 1D). A clear GFP signal was detected in the whole leaf, crude nuclei, purified nuclei, and FAPS-sorted particles from NbCoilinA-GFP-expressing samples but was absent in all corresponding NC samples. Notably, the isolated GFP-positive particles could be recovered by ultracentrifugation, and the GFP signal intensity in these fractions was comparable to that of purified nuclei. In contrast, the nuclear protein histone H3 was significantly depleted in the isolated particles but enriched in the ‘clean’ nuclei sample, indicating that the method specifically enriches for NbCoilinA-GFP-positive particles. To evaluate contamination from other organelles, immunoblots were also performed using anti-Actin and anti-BiP antibodies, recognizing cytoplasm and ER proteins, respectively. Both markers were enriched in leaf samples but barely detectable in nuclei or FAPS-sorted particles. These results indicate that the isolation protocol effectively removes cytoplasmic and ER contaminants, yielding highly purified CB-like structures. Confocal microscopy confirmed that FANS purified nuclei remained intact, and the GFP signal was unaltered, indicating that the structure of CBs was successfully preserved (Fig. 1E) as following FAPS, small round GFP-positive particles, consistent with the expected size (0.15–2 µm) and morphology of CBs, were observed (Fig. 1F).

### Proteomic characterization of plant CBs

**Figure 2.**
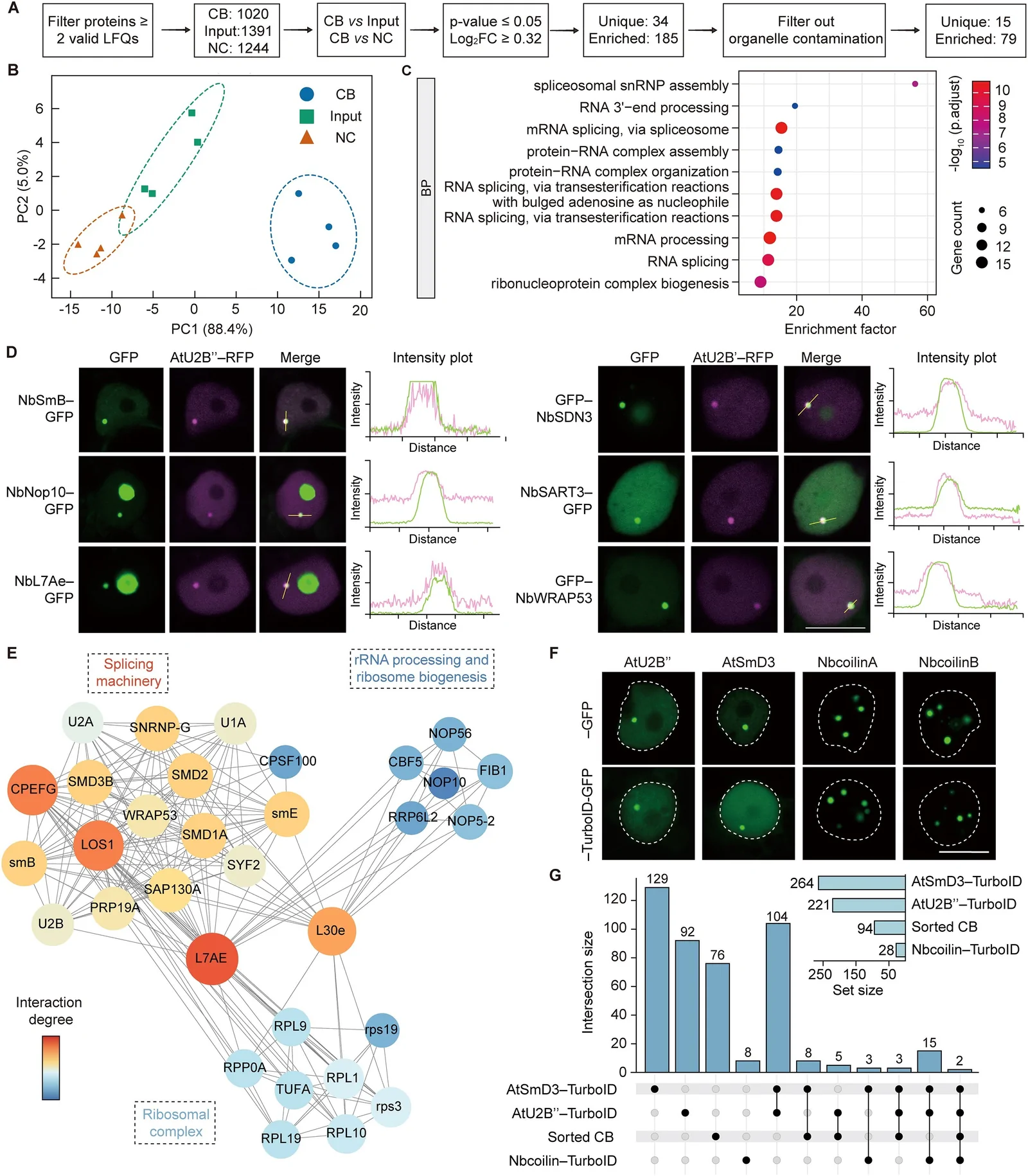
Analysis of the CB proteome. (**A**) Workflow summary of proteomic analysis to identify proteins enriched in CBs compared to input or NC. LFQ values were used to identify proteins enriched in CB samples. (**B**) Principal component analysis (PCA) based on LFQ intensities of proteins detected in samples of FANS-FAPS isolated CBs, input, and NC. Proteins with LFQ values > 0 in at least two replicates per group were retained. Data were *z*-score-normalized before analysis. (**C**) Top 10 GO terms Biological Process (BP) enriched among FANS-FAPS isolated CB proteins. (**D**) Representative confocal images showing localization of identified candidate proteins fused to GFP. AtU2B''-RFP is used as CB marker. Line plots show fluorescence intensity profiles across the CB. Scale bars = 10 μm. (**E**) Protein–protein interaction network of 94 FANS-FAPS isolated CB proteins (from panel A), generated using STRING and visualized in Cytoscape. Edges represent experimentally validated interactions. Disconnected nodes were hidden. (**F**) Subcellular localization of selected CB markers fused to TurboID-GFP in *N. benthamiana* leaves at 40 h post-agroinfiltration. Scale bar = 10 μm. (**G**) Overlap analysis of proteins identified by FANS-FAPS and PL assays using *N.benthamiana* Niben101 gene IDs. Data are based on three independent biological replicates.

CB-like GFP-positive particles isolated from *N. benthamiana* leaves were subjected to LC-MS/MS analysis, alongside nuclear fractions from the same samples (input) and from NC (see ‘Materials and methods’ section for details). LFQ values from the input and NC fractions were used as background to identify proteins specifically enriched in the isolated CBs following the filtering method described in Fig. 2A. Due to the high abundance and fragility of chloroplasts and mitochondria in plant tissue, several plastid- and mitochondria-associated proteins were detected in the dataset, likely as carry-over contaminants released during nuclei isolation and sonication. Therefore, proteins annotated as chloroplast- or mitochondria-localized based on GO annotation were excluded from downstream analyses.

PCA was performed on the unfiltered proteomic data to gain an initial overview of the dataset and assess differences among sample groups. This analysis revealed clear separation between CB, input, and NC samples, each represented by four independent biological replicates (Fig. 2B). The first two principal components, PC1 and PC2, accounted for 88.4% and 5.0% of the total variance, respectively. CB samples clustered distinctly from both input and NC, which showed greater similarity to each other. Pearson correlation analysis showed high intra-group correlation among the four biological replicates of each group (*r* = 0.94–1.00), indicating strong reproducibility (Supplementary Fig. S2B). In contrast, lower inter-group correlations (*r* = 0.78–0.91), particularly between CB and either input or NC, further support the distinct proteomic composition of the CB compared to the nuclear fraction.

**Figure 3.**
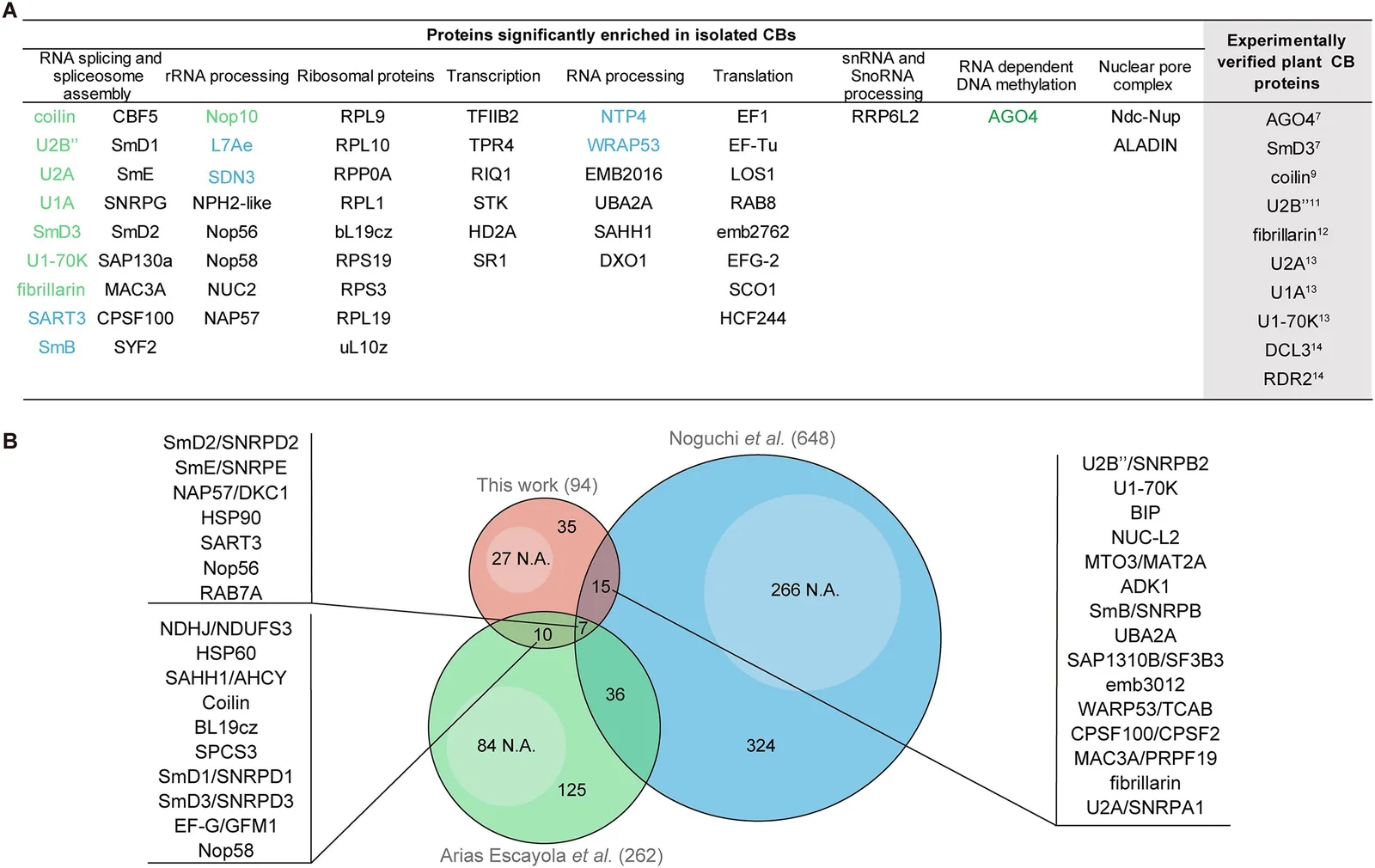
Conservation of CB proteins across kingdoms. (**A**) Functional categorization of proteins identified in FANS-FAPS-isolated plant CBs. Proteins labelled in green were previously reported as plant CB proteins, while those in blue have been experimentally confirmed as CB proteins in this work. (**B**) Comparative analysis of overlapping proteins identified across three studies: N.A. indicates no ortholog was found in *A. thaliana* or *Homo sapiens*, respectively.

A total of 94 proteins were identified in the FANS-FAPS isolated CB-like GFP-positive particles. Fifteen were uniquely detected, while an additional 79 were significantly enriched compared to input or NC, with coilin being the most highly enriched (Supplementary Fig. S2C and D; see the full list in Supplementary Table S1). ‘Unique proteins’ might exclusively localize to the CB or be present in the total nuclear fraction in undetectable amounts. Of the 11 plant CB proteins experimentally confirmed in previous studies [8, 11–15], we successfully identified 9 as enriched in our dataset, namely Coilin, U2B’’, U2A, U1A, SmD3, U1-70K, Nop10, fibrillarin, and AGO4, underscoring the robustness and specificity of the obtained CB proteome (Supplementary Fig. S2C and D; Fig. 3A). The two undetected plant proteins previously described as CB-localized are DCL3 and RDR2, components of the RdDM pathway like AGO4, and shown to overlap with Coilin in 82% and 83% of nuclei in *A. thaliana* protoplasts in immunolocalization experiments, suggesting a dynamic CB localization [15]. Importantly, *DCL3* and *RDR2* exhibit expression levels ~10 times lower than *AGO4* in *N. benthamiana* leaf tissue [16], which could explain their absence from the CB proteome obtained here. In addition, the *N. benthamiana* orthologs of several CB proteins previously characterized in mammalian systems but not yet reported in plants were also detected, including SmB, SART3, WRAP53, Nop58, Nop56, and TFII [17–21].

To assess the quality of our dataset, we selected eight proteins from this list for subcellular localization analysis. These included two well-characterized plant CB components, U2A and Nop10; three proteins previously reported as CB components in mammalian systems but not in plants, namely SmB, SART3, and WRAP53; and three uncharacterized candidates: SDN3, NTP4, and L7Ae. Confocal microscopy revealed that all eight proteins localized to distinct nuclear foci that overlapped with the CB marker AtU2B’’-RFP, confirming their localization to this compartment (Fig. 2D and Supplementary Fig. S2H). To further evaluate purification specificity, we also examined several proteins enriched in the Input/NC fractions, including RBM1, SKP1, and TAF15. These proteins did not localize to NbCoilinA-labelled CBs (Supplementary Fig. S4A). In addition, we compared CBs with markers for other nuclear MLOs, including PhyB photobodies and dicing bodies (SPL1, SUL7, SASF4, and CWF19), and observed distinct localization patterns with minimal overlap (Supplementary Fig. S4B and C). Together, these observations support that the FANS-FAPS strategy preferentially enriches NbCoilinA-associated structures rather than general nucleoplasmic components or other nuclear MLOs.

Due to limited functional annotation of the *N. benthamiana* genome, the *A. thaliana* orthologs of identified CB proteins were used to perform functional enrichment analyses. GO enrichment analysis using the BP ontology revealed strong enrichment for terms such as ‘spliceosome assembly’, ‘RNA splicing’, and ‘ribonucleoprotein complex assembly’ (Fig. 2C), in line with the known functions of the CB. Overrepresented cellular component (CC) terms were topped by ‘CB’ and ‘snRNP complex’, while those in the molecular function (MF) ontology included ‘snRNA binding’ and ‘snoRNA binding’ as the most highly enriched (Supplementary Fig. S2E and F). In contrast, proteins predominantly enriched in the input and NC fractions were associated with more general nuclear functions (full list available in Supplementary Tables S2 and S4), with a significant enrichment for ‘nucleocytoplasmic transport’, ‘gene expression’, and ‘chromatin organization’ (BP), ‘nucleoplasm’, ‘chromatin’, and ‘chromosome’ (CC), and ‘RNA helicase activity’ and ‘chromatin binding’ (MF) (Supplementary Fig. S2G). These findings highlight a clear functional distinction between the nuclear background and the CB-specific proteome. In agreement with the results of the functional enrichment analyses, protein interaction network analysis on identified CB proteins, based on experimentally validated interactions from the STRING database [22], revealed three main protein clusters, related to the splicing machinery, ribosomal complexes, and ribosomal RNA processing, respectively (Fig. 2E), with two ribosomal proteins, L7Ae and L30e, connecting the three groups.

Given the previous use of the CB scaffold protein Coilin as a bait in proximity-labelling studies of animal CBs [4–6], we performed TurboID-based PLs to complement the proteomic dataset obtained from FANS-FAPS isolated CBs. NbCoilinA, the *N. benthamiana* ortholog of the CB scaffold protein Coilin, and its paralog NbCoilinB were used as baits. Two additional characterized plant CB proteins involved in distinct small nuclear ribonucleoprotein complexes, AtU2B″, a component of the U2 snRNP [23], and AtSmD3, a member of the Sm core [24], were also included. Each protein was fused to either GFP or GFP-TurboID to assess the localization of the resulting proteins. Both GFP and GFP-TurboID fusion proteins localized to CBs (Fig. 2F). Notably, when expressed under the strong 35S promoter, both NbCoilinA-GFP/GFP-TurboID and NbCoilinB-GFP/GFP-TurboID formed multiple nuclear foci yet maintained highly specific localization with no detectable signal in the nucleoplasm, suggesting that these scaffolding proteins are limiting factors for CB assembly. PL assays were performed using TurboID-tagged, GFP-free versions of the four bait proteins. Compared to AtU2B'' and AtSmD3, NbCoilinA and NbCoilinB exhibited lower accumulation levels; nevertheless, all fusion proteins displayed biotinylation activity (Supplementary Fig. S5A). PL identified 264 proteins labelled by AtSmD3-TurboID, 221 by AtU2B''-TurboID, and 28 by Nbcoilin-TurboID (NbcoilinA + NbcoilinB) (Fig. 2G, full list available in Supplementary Table S3). Functional enrichment analysis revealed distinct profiles (Supplementary Fig. S5B–D): proteins labelled by AtU2B″-TurboID were enriched in ‘ribosomal RNA processing’ and ‘RNA splicing’ (BP), ‘ribosomal structural constituents’ (MF), and ‘ribosomal protein’ (CC); AtSmD3-TurboID-labelled proteins showed enrichment in ‘mRNA processing’ and ‘non-membrane organelle assembly’ (BP), ‘single-stranded RNA binding’ (MF), and ‘ribosomal protein’ (CC); NbCoilin-TurboID-labelled proteins were associated with ‘centromere complex assembly’ (BP), ‘structural molecule activity’ (MF), and ‘ribosomal protein’ (CC). These results highlight the bait-dependent nature of proximal proteomes, despite all baits localizing to the same MLO. A total of 18 proteins overlapped between the proximity-labelling datasets and the FNAS-FAPS isolated CB proteome (Fig. 2G). GO enrichment analysis of these proteins revealed a significant overrepresentation of ‘splicing’ (BP), ‘poly(A) RNA binding’ (MF), and ‘nuclear body’ (CC) categories (Supplementary Fig. S5E).

A significant overlap was observed between the AtSmD3- and AtU2B’’-TurboID datasets, with 104 shared proteins (Fig. 2G). GO analysis of these overlapping proteins (Supplementary Table S5) indicated an enrichment in general nuclear functions such as ‘translation’ and ‘gene expression’ (BP) (Full list available in the Supplementary Table S6). Although 13 of these 104 proteins were associated with splicing, a known function of CB [7, 25], the overall profile suggested a broader nuclear labelling pattern. This broader labelling may be attributed to the observed partial localization of AtU2B’’ and AtSmD3 in the nucleoplasm (Fig. 2F), which could allow labelling of non-CB proximal proteins.

### Proteomic comparison between plant and animal CBs

We next compared the FANS-FAPS isolated plant CB proteome to the human cell CB proximity proteomes produced by Arias Escayola *et al*. (2025) and Noguchi *et al*. (2024) (Fig. 3B). Orthology between *Arabidopsis* and human proteins was assessed using reciprocal BLAST analysis (RBH) with thresholds of pairwise identity ≥ 30%, *E*-value ≤ 10^−10^, and query coverage ≥50%, combined with conserved domain architecture, functional annotation, and literature-supported ortholog assessment. Based on this analysis, proteins were classified as ‘Yes (strict RBH)’, ‘Likely (annotation/domain support)’, or ‘No/unclear’. (full list available in Supplementary Table S8). Conserved CB proteins across kingdoms include Sm proteins, U2 RNPs, Nop56/58, fibrillarin, SART3, NAP57, and WRAP53. However, based on this approach, some CB proteins were missing orthologs in the other lineage. Among the 94 proteins identified in this study, 27 had no identifiable human orthologs, including the experimentally validated CB proteins NPT4 and SDN3 (Fig. 2D and Supplementary Fig. S2H), which play a role in RNA processing. Similarly, 266 out of 648 proteins identified by Noguchi *et al*. (2024) and 84 out of 262 proteins identified by Arias Escayola *et al*. (2025) lack orthologs in *A. thaliana*. Among them, TOE1 [4], IRF2BP1 [4], TDP-43 [5] and RIF1 [5] were experimentally confirmed as CB-localized. Notably, TOE1, which participates in RNA 3′ end processing and snRNA maturation, lacks a confident plant ortholog. Conversely, for several CB-associated proteins identified in our work, including NTP4 and SDN3, involved in small RNA 3′ end modification, no clear human orthologs were identified. Together, these observations suggest that while RNA 3′ end regulation may represent a conserved functional feature of CBs, the specific molecular machineries associated with this process may have diversified across kingdoms, potentially reflecting adaptation to lineage-specific RNA regulatory pathways.

## Discussion

Here, we present a FANS-FAPS-based isolation of CBs in the model plant *N. benthamiana*. The method developed in this study shows strong potential for broader applicability across diverse organisms and MLOs, both in plants and animals. The foundational principle, using fluorescently tagged markers coupled with FAPS, is adaptable by simply switching the marker protein. Optimization of buffer compositions and sorting conditions is likely to allow the effective application of this method to a diversity of cells and condensates.

To conduct an effective MLO isolation through FAPS, the selection of an appropriate sorting marker protein is a crucial step. Coilin was chosen for CB isolation because it structurally defines this compartment and displays highly specific localization. Previous studies demonstrated that Coilin knock-out or knock-down directly results in the reduction of CBs in both animals and plants [12, 26–28] and significantly reduces the accumulation of other CB proteins, such as AGO4 [29], or causes mislocalization of other components, including U-RNPs [30], emphasizing Coilin’s essential role in CB formation and maintenance. In addition, expression level of the marker protein represents an important consideration for MLO isolation, as overexpression of scaffold proteins can potentially alter condensate number, size, or composition (Supplementary Fig. S1C). While NbCoilinA-GFP expressed from its native and the pRPS5A promoters exhibited similar localization patterns and CB morphology, 35S promoter-driven expression resulted in altered CB number and distribution (Supplementary Fig. S1). Therefore, an ideal MLO marker should be not only localized specifically to the target compartment and functionally involved in its assembly or activity, but also minimally perturb the native properties of the condensate, ensuring that the isolated particles represent *bona fide* and biologically relevant organelles. In future studies, endogenous tagging of NbCoilinA with a fluorescent tag would further minimize potential perturbations associated with exogenous expression and represents a promising strategy for characterizing native MLO proteomes.

PL has emerged as a powerful tool for analyzing partial MLO proteomes by using engineered enzymes, such as BioID, APEX, or TurboID, when fused to known condensate-resident proteins. Limitations of this approach include a small effective labelling radius (10 nm) compared to typical MLO dimensions (0–5 µm); additionally, the water-soluble nature of biotin may hinder effective diffusion into dense, phase-separated MLOs. In this work, PL of CBs using different resident proteins as baits revealed limited overlap among datasets, highlighting the strong dependence of proteomic outputs on the choice of marker and the partial nature of the resulting proteomes. At the same time, because MLOs are membraneless and highly dynamic, many proteins may associate only transiently with these compartments. Consequently, PL approaches, which capture proteins based on spatial proximity, are well suited for identifying such components. In contrast, the CB proteome obtained via FANS-FAPS represents a high-confidence dataset with reduced nucleoplasmic background, as evidenced by strong overlap with known plant CB proteins, localization and functional GO analysis. Because FANS-FAPS relies on physical isolation of intact condensates rather than bait-dependent labelling, it enables the bait-independent recovery of biochemically stable CB-associated components. However, weakly or transiently associated proteins may dissociate during purification despite formaldehyde crosslinking, potentially explaining why PL approaches identified a larger number of CB-associated proteins. Conversely, FANS-FAPS likely enriches more stably associated condensate components that remain physically linked throughout purification. Together, these observations suggest that PL and FANS-FAPS isolation capture complementary aspects of MLO organization and composition. While PLs are powerful for identifying transient and spatially proximal interactions, FANS-FAPS enriches structurally preserved and stably associated condensate components. Combining these approaches may therefore further improve the specificity, coverage, and resolution of MLO proteome characterization.

Understanding the dynamics of CBs and other nuclear MLOs is essential for elucidating their biological functions. CBs serve as organized platforms for enzymes and substrates, significantly enhancing specific cellular processes, such as RNA processing, ribosome biogenesis, and telomere maintenance. Mathematical modelling suggests that the presence of four CBs per nucleus could increase snRNP folding efficiency 11-fold [31]. The number and size of CBs vary significantly among cell types, developmental stages, and stress conditions. For instance, variations occur between vegetative and sperm cells in *A. thaliana* pollen [32]. Viral infections such as that by groundnut rosette virus (GRV) can induce CB restructuring into multiple CB-like bodies [33], demonstrating CB plasticity and responsiveness to environmental stimuli, similar to other MLOs such as the nucleolus [34]. The dynamic, membraneless nature of MLOs enable rapid exchange and re-distribution of components in response to environmental or developmental cues. Shared localization of proteins such as AGO4 (CB and AB-body) [8] and fibrillarin (CB and nucleolus) [33] exemplifies this plasticity. Notably, under viral stress, only CB-localized AGO4 interacts with V2 from tomato yellow leaf curl virus, which suppresses the RdDM-mediated viral genome methylation [35]. Similarly, GRV ORF3 reorganizes CBs, causing fusion with nucleoli for viral movement. These findings point to the potential significance of quantitative proteomic changes in CBs across physiological states. By leveraging the ability to isolate intact CBs and systematically analyze their proteomes, it is now possible to investigate how their composition dynamically responds to developmental cues and environmental stress. Concurrent isolation and analysis of other nuclear MLOs will enable comparative approaches, providing insights into the functional interplay between CBs and other nuclear compartments. This, in turn, will advance our understanding of how CBs contribute to nuclear organization and function under diverse physiological contexts. Moreover, such studies will shed light on the biophysical and molecular principles governing MLO formation, the dynamic exchange of their components, and mechanisms that underpin their plasticity and biological relevance.

## Supplementary Material

gkag867_Supplemental_Files

## Data Availability

The mass spectrometry proteomics data have been deposited to the ProteomeXchange Consortium via the PRIDE [43] partner repository with the dataset identifier PXD065938.
