# Supplementary material for: Proteome profiling of plant Cajal bodies uncovers kingdom-specific components
Source: Nucleic Acids Res. 2026 Sep 18;54(17):gkag867. doi: 10.1093/nar/gkag867 (PMC13586619; doi:10.1093/nar/gkag867)
Supplement: Supplementary file 1 [file gkag867_supplemental_files.zip › 02_Supplementary material_Zhou et al._V2.pdf]

SUPPLEMENTARY MATERIAL

Proteome profiling of plant Cajal bodies uncovers kingdom-specific components

Yu Zhou (周誉)[1,2,*], Huang Tan (谭煌)[1], Axel Giudicatti[1], Angela Vicente-Luque[1], Marvin Weis[1], Lena Wehinger[1], Marina Schreidl[1], Irina Droste-Borel[3], Boris Macek[3], Kenneth Wayne Berendzen[1,4,*], Rosa Lozano-Durán[1,4,*,#]

[1]Plant Biochemistry Department, Center for Plant Molecular Biology (ZMBP), Eberhard Karls University Tübingen, D-72076, Germany

[2]College of Life Sciences and Oceanography, Shenzhen University, Shenzhen, China

[3]Proteome Center Tübingen, University of Tübingen, Tübingen, D-72076, Germany

[4]Cluster of Excellence GreenRobust, Eberhard Karls University Tübingen, D-72076, Germany

*Correspondence: yu.zhou@szu.edu.cn; kenneth.berendzen@uni-tuebingen.de; rosa.lozano-duran@uni-tuebingen.de.

[#]Lead contact: rosa.lozano-duran@uni-tuebingen.de

SUPPLEMENTARY FIGURES

Supplementary Figures 1-5

SUPPLEMENTARY TABLES

Supplementary Tables 1-8

SUPPLEMENTARY FIGURES

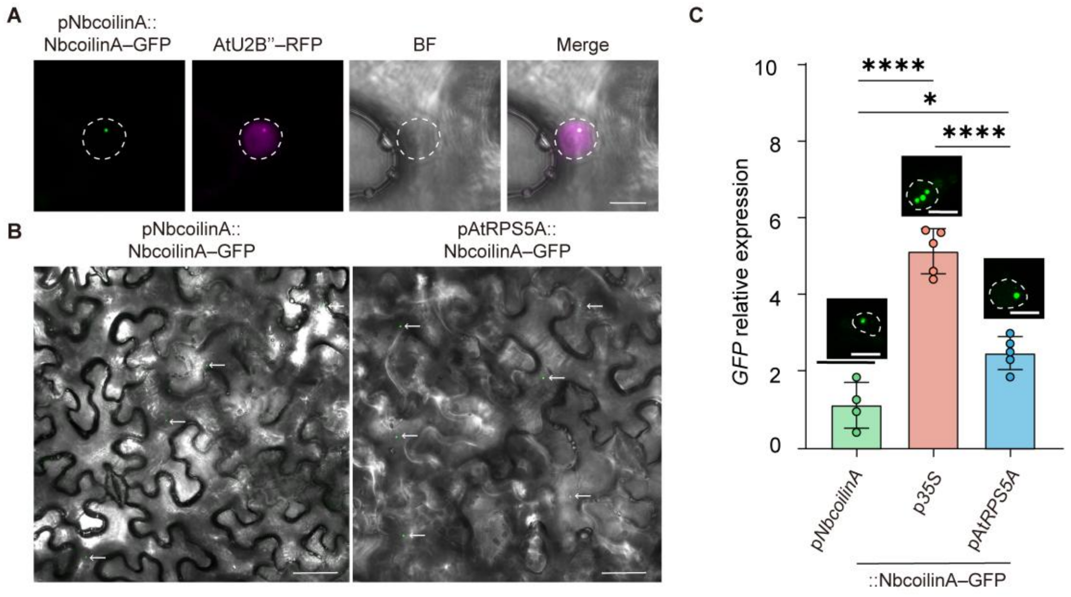

Supplementary Figure 1. Expression pattern of NbCoilinA-GFP under different promoters in *Nicotiana benthamiana*.

A. Co-localization of NbCoilinA-GFP expressed under its native promoter with the CB marker AtU2B″-RFP. Scale bars = 10 μm.

B. Overview of Nbcoilin-GFP expression pattern driven by its native promoter or the pAtRPS5A promoter in *Nicotiana benthamiana* leaf epidermal cells. Scale bars = 10 μm.

C. Comparison of NbCoilinA-GFP transcript levels and CB morphology in leaves expressing NbCoilinA-GFP under its native promoter, the pAtRPS5A promoter, or the 35S promoter. White dashed circles indicate nuclei. Scale bars = 10 μm.

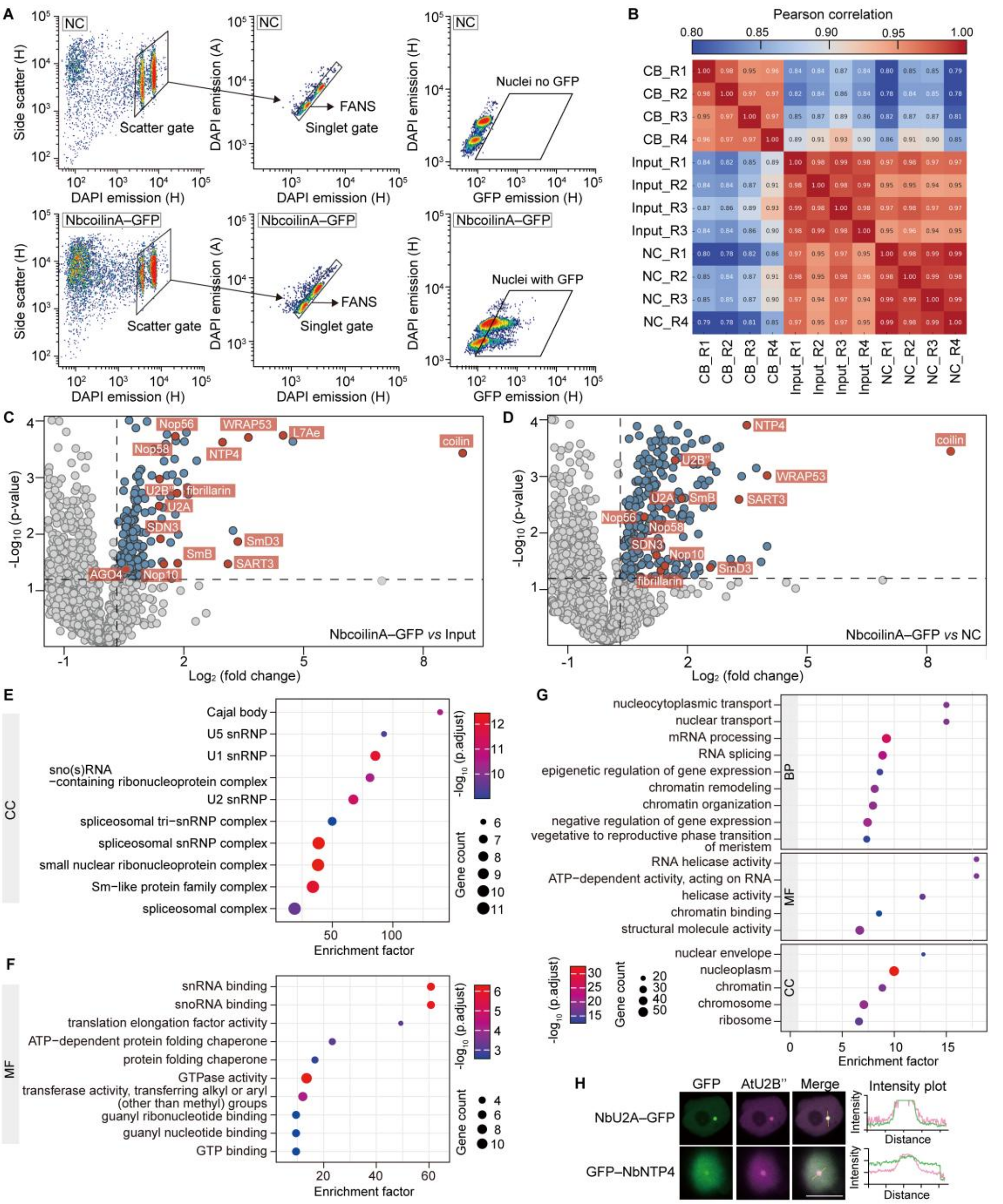

Supplementary Figure 2. Validation and functional enrichment analysis of the sorted Cajal body (CB) proteome.

A. Example gating strategy used for FANS. DAPI-positive nuclei (Scatter gate) were identified based on their side scatter profile, and doublets (Singlet gate) were excluded using DAPI area versus height plots. Both gates were combined (Boolean AND) for nuclei sorting. GFP expression was verified prior to sorting by plotting DAPI versus GFP fluorescence but was not used as a sorting parameter.

B. Pearson correlation matrix of LFQ intensities among biological replicates of CB, input, and NC samples, showing high reproducibility within each sample group. r > 0.9: reproducible technical or biological replicates; r~0.6–0.8: related but distinct interaction profiles.

C, D: Volcano plots illustrating the differential enrichment of proteins in the FANS-FAPS

isolated CB compared to either the input (C) or the NC (D). Proteins with a two-tailed Student's t-test $p < 0.05$ and a fold change greater than 1.25 were considered significantly enriched in the CB.

E. Top 10 Gene Ontology (GO) terms Cellular component (CC) enriched among isolated CB proteins.

F. Top 10 Gene Ontology (GO) terms Molecule function (MF) enriched among isolated CB proteins.

G. GO enrichment analysis of enriched proteins shared between input and NC samples, across biological process (BP), molecular function (MF), and cellular component (CC) categories.

H. Representative confocal images showing localization of identified candidate proteins fused to GFP to the CB. AtU2B''-RFP is used as CB marker. Line plots show fluorescence intensity profiles across the CB. Scale bars = 10 μm.

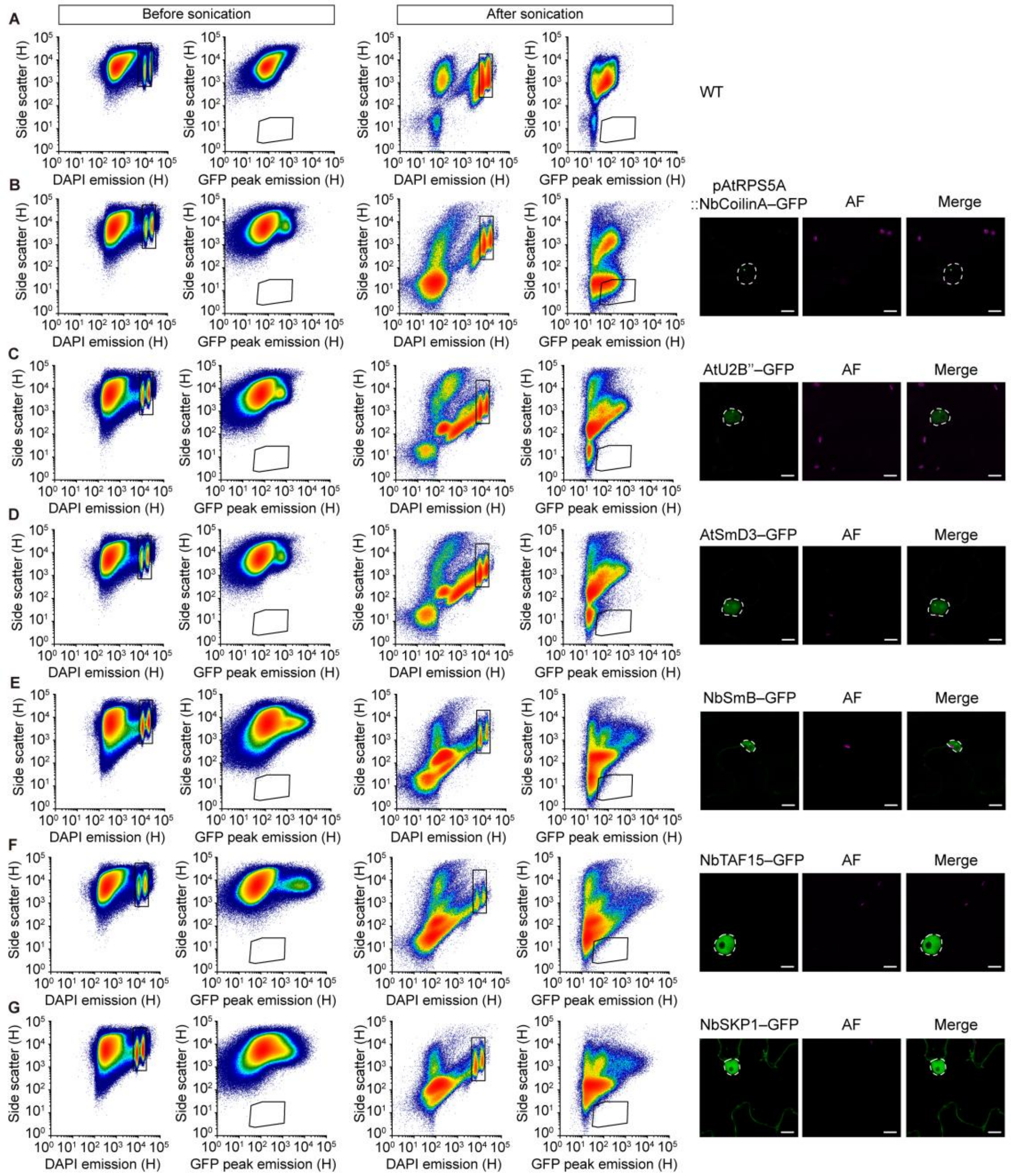

Supplementary Figure 3. Cytometric characterization of different nuclear proteins during FANS-FAPS.

A–G. Cytometry and microscopy profiles of nuclei before sonication and released nuclear particles after sonication from *Nicotiana benthamiana* leaves expressing different GFP fusion nuclear proteins. DAPI fluorescence versus side scatter (SSC) plots were used for nuclei identification (rectangle). DAPI fluorescence SSC and GFP fluorescence versus SSC are shown before and after sonication. FAPS gate indicates the particle populations selected for CB sorting (polygon). Representative fluorescence microscopy images of the corresponding sorted particles are shown on the right. GFP is shown in green and autofluorescence (AF) in magenta. WT control transformed with the empty vector lacking GFP fluorescence. White dashed circles indicate nuclei. Scale bars = 10 µm.

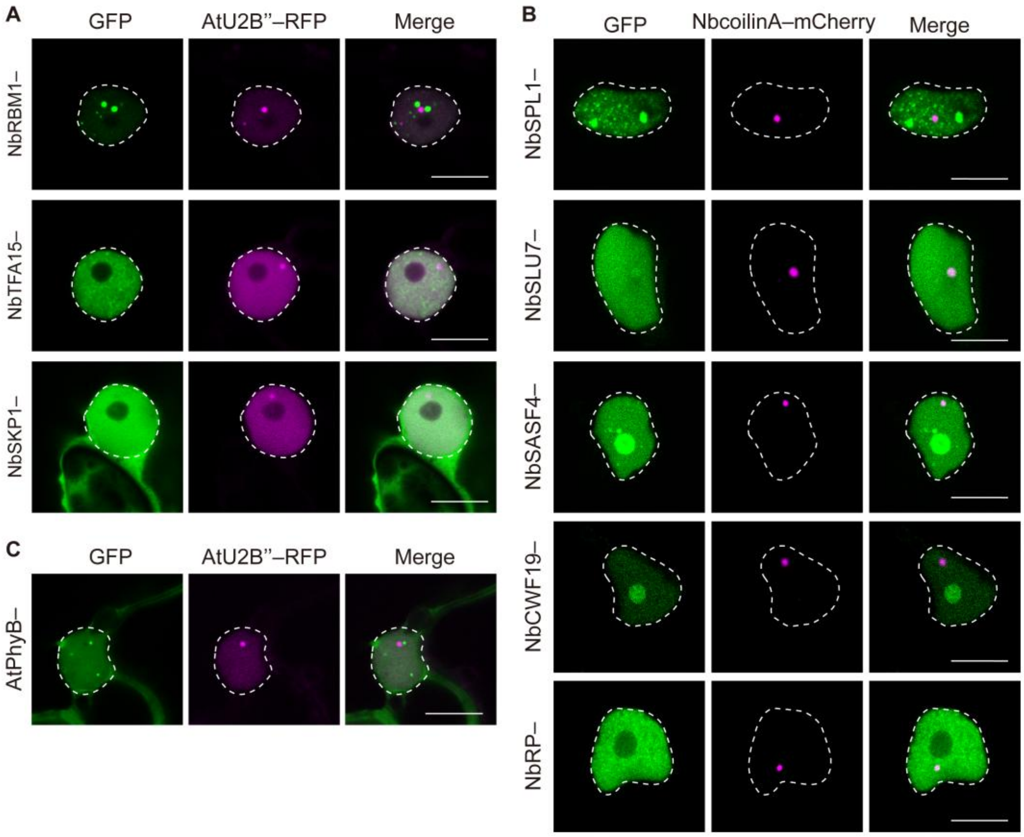

Supplementary Figure 4. Localization of non-CB proteins and other nuclear MLO markers with CB markers in *Nicotiana benthamiana* leaves.

A. Selected proteins enriched in the NC and Input fractions were fused to GFP and co-expressed with the CB marker AtU2B″-RFP. White dashed circles indicate nuclei. Scale bars = 10 μm.
B. Dicing body markers fused to GFP localize to nuclear structures distinct from NbCoilinA-mCherry labelled CBs. Scale bars = 10 μm.
C. AtPhyB-GFP labelled photobodies form nuclear structures spatially separated from AtU2B″-RFP labelled CBs. Scale bars = 10 μm.

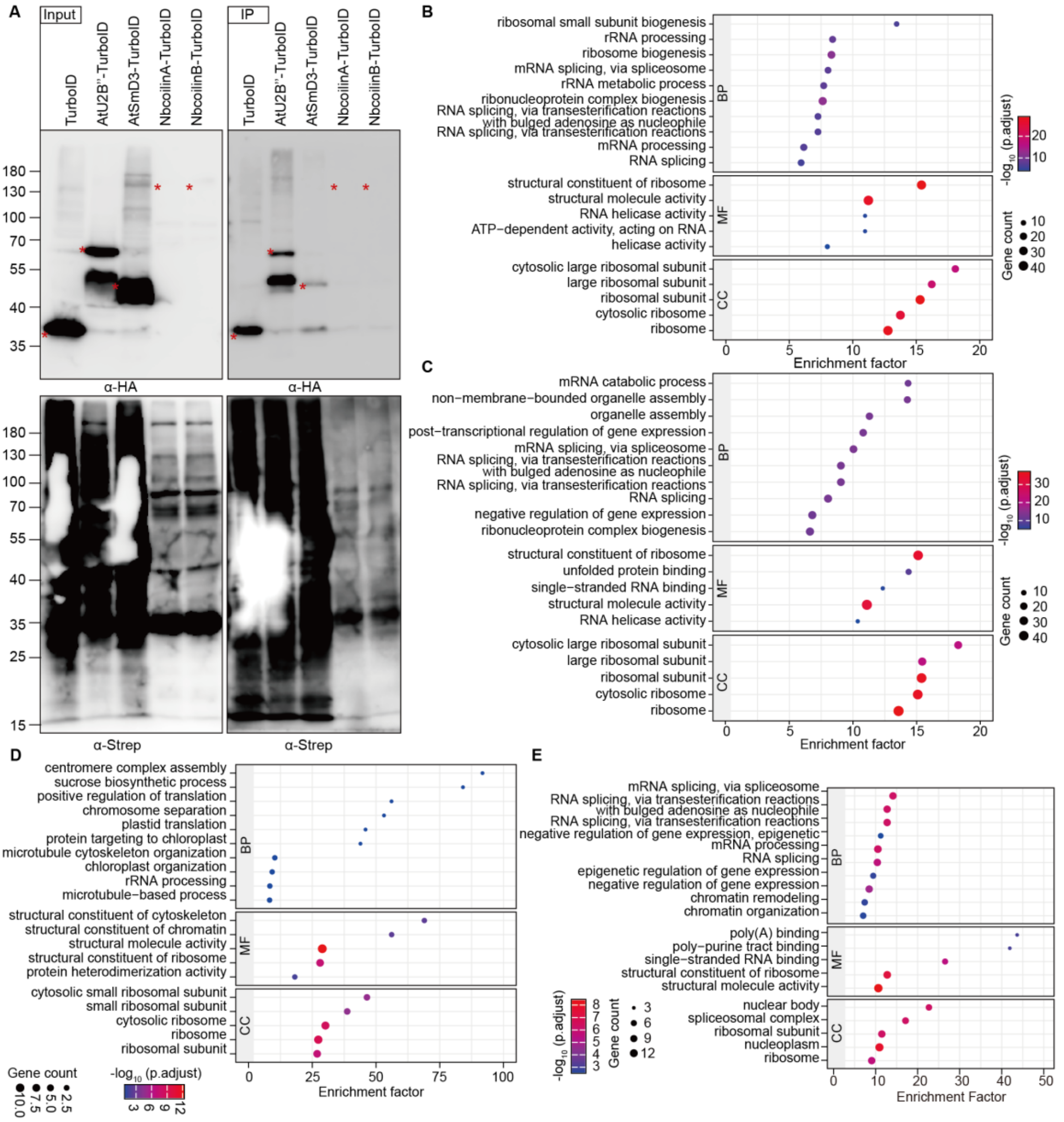

Supplementary Figure 5. TurboID-based proximity labelling assays with CB markers and functional enrichment analysis of identified proteins.

A. TurboID-fused CB markers retain biotinylation activity in *N. benthamiana* leaves in the presence of 50 µM exogenous biotin. Western blot analysis shows accumulation of the fusion proteins (α-HA) and streptavidin-HRP detection of biotinylated proteins from samples expressing AtU2B''-TurboID (61 kDa), AtSmD3-TurboID (49 kDa), NbcoilinA-TurboID (126 kDa), NbcoilinB-TurboID (124 kDa) and TurboID alone (35 kDa). The corresponding fusion protein bands are indicated by red asterisks.

B–E. Gene Ontology (GO) enrichment analyses of proteins identified in TurboID-based proximity labelling assays with the CB markers in A. B, AtU2B''-TurboID; C, AtSmD3-TurboID; D, Nbcoilin-TurboID; E, 22 proteins overlapping between FANS-FAPS isolated CB and TurboID-based proximity labelling. Enrichment results are shown for Biological Process (BP), Molecular Function (MF), and Cellular Component (CC) categories.

SUPPLEMENTARY TABLES

Supplementary Table 1. List of putative plant Cajal body components identified by FANS-FAPS CB isolation.

Supplementary Table 2. Proteins enriched in input and NC nuclear fractions.

Supplementary Table 3. Proteins labelled by AtU2B"-TurboID, AtSmD3-TurboID, and NbCoilin-TurboID, compared to TurboID-only controls.

Supplementary Table 4. Gene ontology analysis of putative Cajal body components (from Supplementary Table 1) and proteins enriched in the nuclear fraction (from Supplementary table 2).

Supplementary Table 5. Gene ontology analysis of proteins overlapping between the indicated datasets.

Supplementary Table 6. Gene ontology analysis of proteins labelled by AtU2B"-TurboID, AtSmD3-TurboID, and NbCoilin-TurboID (from Supplementary Table 3).

Supplementary Table 7. Primers used in this work.

Supplementary Table 8. Cross-kingdom identification of orthologous proteins.
